# Control of T Cell Metabolism by Cytokines and Hormones

**DOI:** 10.3389/fimmu.2021.653605

**Published:** 2021-04-13

**Authors:** Emma L. Bishop, Nancy Gudgeon, Sarah Dimeloe

**Affiliations:** College of Medical and Dental Sciences, Institute of Immunology and Immunotherapy, Institute of Metabolism and Systems Research, University of Birmingham, Birmingham, United Kingdom

**Keywords:** T cell, metabolism, glycolysis, mitochdonrion, cytokine, hormone

## Abstract

Dynamic, coordinated changes in metabolic pathway activity underpin the protective and inflammatory activity of T cells, through provision of energy and biosynthetic precursors for effector functions, as well as direct effects of metabolic enzymes, intermediates and end-products on signaling pathways and transcriptional mechanisms. Consequently, it has become increasingly clear that the metabolic status of the tissue microenvironment directly influences T cell activity, with changes in nutrient and/or metabolite abundance leading to dysfunctional T cell metabolism and interlinked immune function. Emerging evidence now indicates that additional signals are integrated by T cells to determine their overall metabolic phenotype, including those arising from interaction with cytokines and hormones in their environment. The impact of these on T cell metabolism, the mechanisms involved and the pathological implications are discussed in this review article.

## Introduction

T lymphocytes are critical mediators of the adaptive immune response. Following recognition of pathogen- or tumour-derived antigens by the T cell receptor (TCR), together with costimulatory signals provided by antigen-presenting cells, they clonally expand and traffic to tissues. Here, they exert effector functions including the direct killing of infected and malignant cells, and secretion of cytokines to coordinate the immune response. These significant changes in activity occur alongside substantial changes in cellular metabolism. This provides energy and biosynthetic precursors for clonal expansion and the synthesis of effector molecules, as well as having direct effects on T cell signaling, gene transcription and protein translation. The importance of these dynamic metabolic changes for T cell immune function renders these cells highly susceptible to their microenvironment. For example, changes in the abundance of nutrients in diseased tissue environments alter the metabolic capacity of T cells present, leading to dysregulation of their activity. Additionally, accumulating evidence indicates that T cell metabolic capacity can be further fine-tuned through recognition of local and systemic stimuli including cytokines and hormones, which will be the focus of this review.

## Metabolic Reprogramming and T Cell Function

In their resting or quiescent state, T cells rely largely on the oxidative phosphorylation (OXPHOS) of glucose and fatty acids in the mitochondria to generate ATP for homeostatic processes. However, upon antigen recognition their metabolic phenotype alters significantly, shifting towards an anabolic profile, which supports the substantial energetic and synthetic demands of clonal expansion and effector protein production. Among the most marked changes are substantially increased rates of glucose and amino acid uptake. Increased glucose uptake is mediated by trafficking of the Glut1 transporter to the cell surface (1), whereas amino acids, particularly glutamine, influx *via* heightened expression of transporters such as LAT1, SNAT-1, SNAT-2 and ASCT2 (2–4).

Once inside the cell, glucose is converted in the cytoplasm into pyruvate *via* the 11 enzymatic reactions of glycolysis. This also occurs in quiescent cells, however, the fate of glucose-derived pyruvate is altered following T cell activation. Specifically, a substantially larger proportion is reduced to lactate and excreted, rather than being converted into acetyl-CoA for mitochondrial oxidation (1, 5). Despite providing much less ATP per molecule of glucose, this process permits more rapid conversion of glucose to pyruvate, through regeneration of the redox cofactor NAD^+^ and maintenance of favorable AMP/ATP ratios. This ensures abundance of the multiple biosynthetic precursors generated by glycolysis, which support nucleic acid, protein and lipid biosynthesis. Additionally, heightened glycolytic activity directly promotes effector molecule expression, through control of protein translation. Specifically, the glycolytic enzyme glyceraldehyde 3-phosphate dehydrogenase (GAPDH) binds certain mRNAs *via* AU-rich regions in their 3’ UTR, preventing their translation. This mechanism is disengaged in highly-glycolytic, activated T cells permitting the translation of these mRNAs, including that of the key T cell effector cytokine, interferon-gamma (IFN-γ) (6).

Given that T cell activation drives substantial increases in glucose uptake, even despite this general shift towards pyruvate reduction to lactate, a parallel increase in mitochondrial pyruvate oxidation nonetheless occurs (7, 8). Here, pyruvate enters the mitochondria, where it is converted into acetyl-CoA and sequentially oxidized in the tricarboxylic acid (TCA) cycle. This yields carbon dioxide, water and GTP and additionally reduces the electron carriers NADH and FADH_2_. Together these drive activity of the electron transport chain (ETC), which produces ATP and reactive oxygen species (ROS). Like glycolysis, this increased mitochondrial OXPHOS activity also directly supports T cell activation, proliferation and effector function. For example, mitochondrial ROS stabilize transcription factors such as NFAT promoting effector molecule expression (7), whilst TCA cycle intermediates are used for anabolic processes, as well as post-translational modifications of proteins –notably the acetylation of histones, permitting gene transcription (9, 10) and acetylation of enzymes including GAPDH, which augments its function (11). The TCA cycle oxidizes not only glucose in this manner, but also fatty acids and the amino acid glutamine. Glutamine, like glucose, is critically required by proliferating T cells (8). Its roles include promoting activity of the key metabolic kinase mechanistic target of rapamycin (mTOR, see below) (4), and also, following conversion to α-ketoglutarate, directly contributing to the TCA cycle, especially when glucose availability is limiting (8). Consistently, as well as increasing expression of glutamine transporters, activated T cells also upregulate glutaminolytic enzymes (5).

Key signaling pathways driving T cell metabolic reprogramming following antigen encounter include the phosphatidylinositol-3-kinase (Pi3K)/Akt/mTOR axis and myelocytomatosis oncogene (c-Myc) signaling. Notably, co-stimulatory signaling *via* CD28 plays a key role in initiating these pathways (1), however, increased strength of TCR stimulation can also drive metabolic changes in the absence of CD28 signaling (12, 13). Specifically, TCR/CD28 ligation induces Pi3K-dependent phosphorylation of Akt (14). Phospho-AKT (pAkt) then augments glycolysis *via* mechanisms including the promotion of Glut1 trafficking to the cell surface, phosphorylation of glycolytic enzymes and, importantly, activation of the kinase mTOR. This occurs through the phosphorylation of Tuberous Sclerosis Complex-2 (TSC-2), which leads to the proteasomal degradation of this inhibitor and consequent de-repression of mTOR activity (1).

mTOR regulates cellular metabolism through controlling the translation and stability of key metabolic transcription factors. mTOR-deficient T cells fail to engage metabolic reprogramming upon activation and do not proliferate or gain effector functions (15). Notably, however, unbiased proteomic analyses have revealed selectivity of the role of mTOR in T cell metabolism, since treatment of CD8^+^ T cells with the mTOR inhibitor, rapamycin, decreases the abundance of metabolic proteins including glucose transporters and enzymes involved in glycolysis and cholesterol metabolism, but not others associated with mitochondrial OXPHOS and glutamine metabolism (16).

Transcription factors so far identified to play a role eliciting T cell metabolic reprogramming include c-Myc (5); which is upregulated in an Akt- and mTOR-dependent manner and drives increases in the uptake and metabolism of glucose, glutamine and other key amino acids; the Sterol Regulatory Element Binding Proteins 1 and 2 (SREBP1 and 2 (17), which again are mTOR-induced and regulate lipid synthesis pathways, the nuclear receptor estrogen-related receptor-α (ERR-α) (18), IRF4 (19), Foxo1 (20) and EZH2 (21). Hypoxia inducible factor -1α (HIF-1α) expression is also upregulated upon T cell activation, even in oxygen replete conditions. HIF-1α activation drives T cell glycolysis and is required to support their effector function (22).

In addition to these signals downstream of the TCR, co-stimulatory and cytokine receptors (discussed further below) T cell metabolism is further regulated as a consequence of the significant metabolic changes they undergo, in a mechanism previously described as “bottom-up signaling” (23). One example of this is the activation of mTOR by amino acid sensing. This mechanism drives increased activity of mTOR in response to increased abundance of leucine and arginine and may be a key driver of mTOR-dependent metabolic reprogramming in T cells, since expression of the large neutral amino acid transporter, SLC7A5 (which mediates uptake of leucine and arginine among other amino acids) is absolutely required for T cells to become activated and differentiate into effector phenotypes (3). Additionally, T cells substantially upregulate expression of amino acid sensing machinery upon activation, and again this is required for T cell activation and the acquisition of effector function (24).

Another important example of how the metabolic status of T cells influences the signaling pathways driving their differentiation is *via* activity of AMP-activated protein kinase (AMPK). This kinase counterbalances mTOR as a regulator of cellular metabolism. Rising AMP concentrations, indicating energy expenditure, activate AMPK, which in turn promotes ATP production by phosphorylating targets in glycolytic and fatty acid oxidation pathways. It also limits further ATP consumption by inhibiting catabolic processes, protein translation and inhibiting mTOR activity. AMPK plays an important role regulating metabolic plasticity of T cells under nutrient poor conditions. Under glucose-replete conditions, quiescent T cells adopt aerobic glycolysis upon activation. However, an environment with reduced nutrient availability leads to metabolic reprogramming dictated by AMPK activity. Cells engage glutamine-dependent OXPHOS and IFN-γ mRNA translation is suppressed to meet bioenergetic requirements and viability (8).

Whilst the metabolic changes described here are largely observed in all activated T cells, it is becoming increasingly clear that functionally-distinct T cell subsets demonstrate different metabolic phenotypes and rely on distinct metabolic pathways to exert their specialist immune functions. Such differences were first characterized by comparing the metabolic phenotype of naïve and memory T cells. These studies identified that memory T cells are metabolically primed for their role surveying tissues for antigen recurrence and responding rapidly to this. For example, they contain more complex mitochondria than naïve cells, which support increased survival under hypoxia (25) and greater longevity *in vivo* (26). Additionally, this increased mitochondrial capacity, together with their swift adoption of glycolysis upon activation (supported by increased cytosolic glycolytic enzyme abundance) support their rapid proliferation and cytokine production upon TCR ligation (26–28). Subsequent studies have found that pro-inflammatory Th1, Th2 and Th17 subsets of helper CD4+ T cells are metabolically distinct from regulatory CD4+ T cells, which control inflammatory immune responses. For example, Th1 and Th17 cells are reported to be highly glycolytic (29, 30), and Th17 cells to demonstrate high capacity for glutamine metabolism (31) whereas regulatory T cells (TReg) appear to tolerate low glucose availability but rely on fatty acid oxidation for their suppressive function (32, 33).

The significant metabolic demands of activated T cells to support their immune function means that they are highly susceptible to the metabolic features of their microenvironment. For example, decreased availability of oxygen, glucose and amino acids in the tumor microenvironment can impair T cell function, whilst conversely the build-up of metabolic intermediates and end-products in malignant and inflamed tissue also alter T cell activity. These factors have already been reviewed by ourselves and others (22, 34) and will not be the focus of this article. Rather we will discuss the emerging evidence that other factors present at altered abundance in pathology, either systemically or locally in diseased tissue also impact T cell metabolism. Specifically, we will focus our attention on how T cell metabolism is controlled by the activity of cytokines and hormones.

## Impact of Cytokines on T Cell Metabolism

It is well known that cytokines and their signaling pathways exert potent effects on T cell activation, differentiation, and function. However, the impact of cytokines on T cell metabolism, particularly in the context of disease, remains less clear. Some studies have begun to explore the potential role of cytokine-mediated metabolic changes in controlling T cell function, but further in-depth investigations are required to understand the mechanisms and relevance of these effects in disease (**Table 1**).

**Table 1:** Effects of cytokines + hormones on T cell metabolism.

**Cytokine**	**Effects on T cell metabolism**	**Refs**
IL-2	• Augments Pi3k/Akt/mTOR activity in CD4^+^ (Th1) and CD8^+^ T cells • Drives SLC7A5 expression in CD4+ and CD8+ T cells • Increases c-Myc expression in CD4+ and CD8+ T cells • Augments PDK-mediated mTOR activition in CD8+ T cells • Sustains glycolysis, mitochondrial function and lipid biosynthesis in TReg	(35, 36) (3, 13, 35) (13) (37, 38) (39, 40)
IL-7	• Sustains/augments glycolysis in total CD3+ T cells *via* STAT5-mediated Akt induction • Induces Glut1 expression in Tscm CD8+ T cells • Increases glycerol uptake for triglyceride synthesis in CD8+ memory T cells	(41, 42) (43) (28)
IL-15	• Increases CPT1a expression and drives mitochondrial fusion and cristae remodeling in CD8+ memory T cells • Promotes expression of antioxidant molecules in CD8+ T cells • Increases mTOR activity in memory CD8+ T cells	(26, 44) (45, 46) (47, 48)
IL-21	• Promotes mitochondrial biogenesis and FAO in total CD3+ T cells and CD8+T cells • Promotes mTOR activity in TRegs.	(49, 50) (51)
IFN-γ	• Activates mTOR *via* STAT1 in CD8+ T cells under weak TCR stimulation	(52)
IL-1β	• Activates mTOR and HIF-1α and drives glycolysis in total CD4+ T cells and Th17/iTReg	(53–55)
IL-23	• Promotes mTOR actvitiy in Th17 cells • Increases PKM2 phosphorylation • Together with IL-1β, drives metabolic reprogramming in Th17 cells	(56) (57) (58)
TNF-α	• May increase expression of metabolic genes including HK2, PKM2 and TNFAIP3 in total and memory CD4+ T cells	(59, 60)
TGF-β	• Inhibits mTOR activity in CD4+ T cells *via* Smad3-mediated inhibition of Pi3K/Akt • Increases mitochondrial capacity and inhibits glycolysis in TReg • Inhibits mitochondrial complex V activity in memory CD4+ T cells	(61) (29, 61–63) (63)
**Hormone**	**Effects on T cell metabolism**	**Refs**
Leptin	• Drives Glut1 expression, glucose uptake and metabolism in total CD3+ T cells, and particularly CD4+ Th17 T cells, but not TReg • Increases mitochondrial mass and respiratory capacity of CD8+ T cells *in vitro* and within tumors • Decreases CD8+ T cell glycolysis and increases CPT1a expression and FAO *via* STAT3	(64, 65) (66) (67)
Adiponectin	• Suppresses glycolysis in CD4+ Th1 T cells	(68)
Insulin	• Augments c-Myc, glucose transporter and glycolytic enzyme expression in CD4+ and CD8+ T cells	(69)
Glucocorticoids/ Corticosteroids	• Suppress FAO and mitochondrial membrane potential in low-affinity memory CD8+ T cells	(70)
Vitamin D	• Decreases expression of Pi3k/Akt/mTOR pathway components and c-Myc in CD4+ T cells • Decreases mTOR activity in CD4+ T cells • Increases methionine synthesis and consequent DNA methylation in CD4+ T cells	(71–73) (74) (75)

### Cytokines Involved in T Cell Activation and Homeostasis

The majority of studies in the area to date have focused on the common receptor γ chain (γc) cytokine family, including IL-2, IL-7, IL-15, and IL-21 (76). Each of these cytokines has unique roles in regulating the changes in metabolism required at different stages of a T cell response. The actions of the γc cytokines are coordinated largely through their induction of three main signaling pathways, Pi3K/Akt, JAK/STAT, and MEK/ERK (76).

#### IL-2

**Figure 1:**
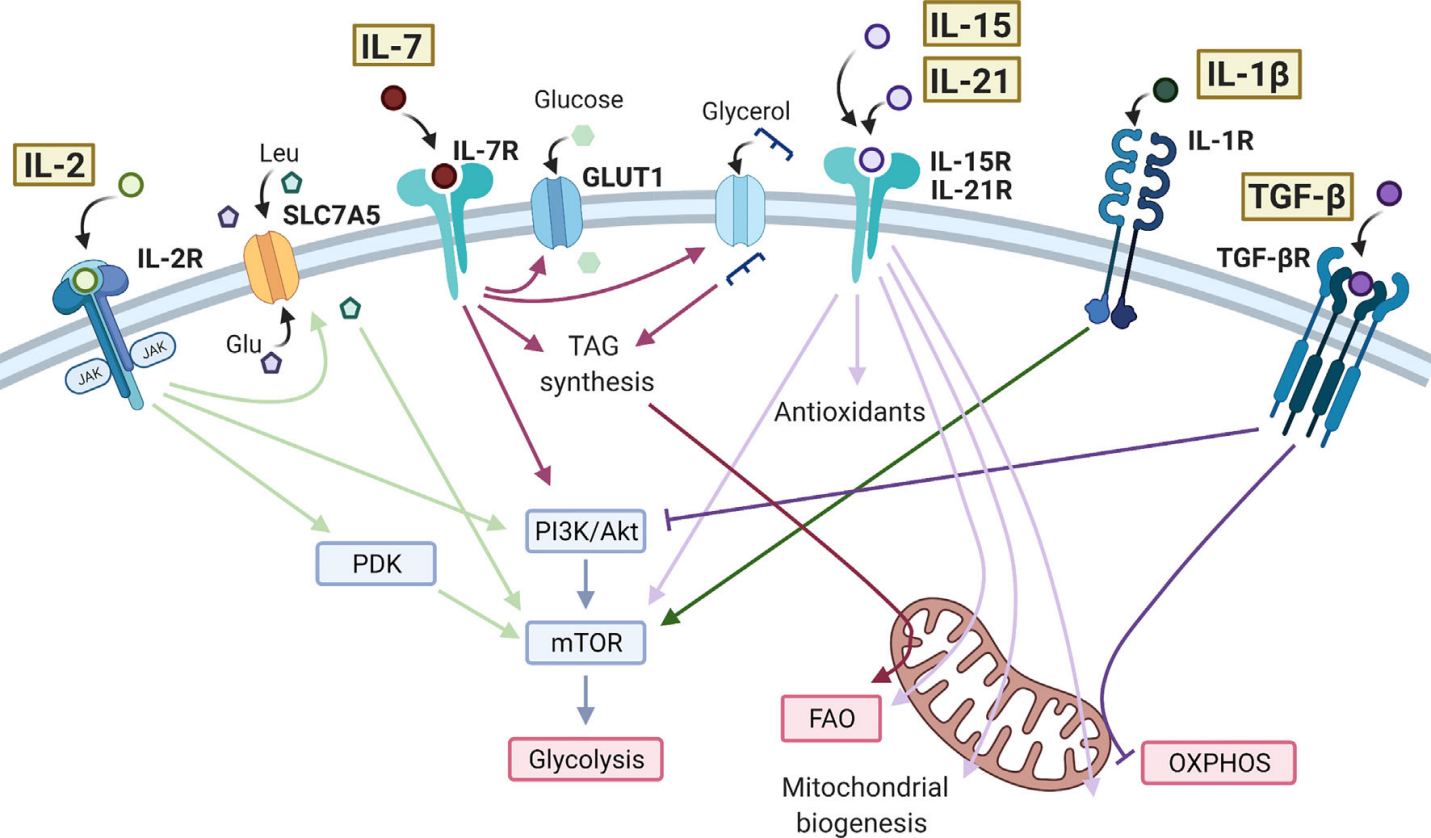
Effects of cytokine signaling on T cell metabolism. IL-2 bound to the IL-2 receptor (IL-2R) is able to promote the activity of mTOR through PDK in CD8^+^ T cells and PI3K/Akt in CD4^+^ T cells. IL-2 promotes the expression of SLC7A5 at the cell surface leading to influx of amino acids, further promoting the activity of mTOR and glycolysis. IL-7 also signals through the PI3K/Akt/mTOR pathway, as well as promoting GLUT1 expression to sustain glycolysis. In CD8^+^ memory T cells, IL-7 increases glycerol uptake and triacylglycerol (TAG) synthesis to enhance fatty acid oxidation (FAO). IL-15 and IL-21 have very similar action in promoting antioxidant production, FAO, mitochondrial biogenesis, and oxidative phosphorylation (OXPHOS) in T cells. IL-15 and IL-21 have also been shown to increase mTOR activity in CD8^+^ memory T cells and Tregs, respectively. IL-1β drives mTOR activity and glycolysis in CD4^+^ T cells, typically promoting a Th17 cell phenotype. The anti-inflammatory cytokine TGF-β inhibits glycolysis in CD4^+^ T cells via the PI3K/Akt/mTOR pathway and inhibits OXPHOS in CD4^+^ memory T cells. Created with Biorender.com.

IL-2 is essential for the upregulation of glycolysis upon TCR engagement, mediated mainly through activation of mTOR downstream of Pi3K/Akt signaling (35, 36) **Figure 1**. IL-2 signaling in T cells also induces expression of the amino acid transporter, SLC7A5 (3, 13). As described above, uptake of amino acids is essential for sustained mTOR activity and control of c-Myc expression in activated T cells. Mechanisms in CD8^+^ cytotoxic T cells (CTLs) appear to be slightly different, with IL-2-induced metabolic reprogramming occurring independently of Pi3K/Akt signaling and instead requiring pyruvate dehydrogenase kinase (PDK)-mediated mTOR upregulation (37, 38). The role of IL-2 in regulating T cell metabolism may be particularly important in TReg cells, which abundantly express the high-affinity IL-2 receptor, CD25 and require IL-2 signaling to maintain their phenotype and function (77). In these cells it has been shown that sustained glycolysis though IL-2-dependent Pi3K/Akt/mTOR signaling is essential for their suppressive activity (39) and additionally that Tregs lacking the IL-2 receptor (CD25) exhibit mitochondrial dysfunction and impaired lipid biosynthesis, leading to apoptosis (40).

#### IL-7, IL-15, and IL-21

IL-7 is crucial for the metabolic homeostasis of T cells, maintaining glycolysis by controlling expression of Glut1 and the glycolytic enzyme hexokinase 2 (HK2). Both the Pi3K/Akt and JAK/STAT signaling pathways are implicated here, with JAK recruitment to the IL-7Rα chain eliciting STAT5 phosphorylation which, in turn transcriptionally induces Akt expression to amplify Pi3K/Akt pathway activity (41, 42) (**Figure 1**). CD8^+^ stem memory T (Tscm) cells are a unique subset with high self-renewal capacity and persistence in autoimmunity. Tscm cells are characterized by high levels of IL-7-induced Glut1 expression but low levels of lactate export, suggesting that in these cells, pyruvate is preferentially being oxidized in the mitochondria (43). IL-7 also promotes long-term survival of CD8^+^ memory T cells by upregulating glycerol uptake for triglyceride synthesis and storage to provide substrate for fatty acid oxidation (FAO) (28).

Also essential for memory T cell development, IL-15 drives mitochondrial fusion, cristae remodeling and increases mitochondrial spare respiratory capacity (SRC) of memory CD8+ T cells. This is associated with upregulation of carnitine O-palmitoyltransferase 1 (CPT1a), which catalyses the transfer of the acyl group of a long-chain fatty acyl-CoA from coenzyme A to l-carnitine, thereby supporting FAO (26, 44) **Figure 1**. In one study, it was also seen that the addition of IFN-α, an important cytokine in viral infection and an inducer of IL-15, to IL-15-driven CD8^+^ memory T cells could further increase SRC (78). In parallel to these mitochondrial changes, IL-15 also promotes expression of antioxidant molecules in CD8+ T cells including glutathione reductase, thioredoxin reductase 1, peroxiredoxin and superoxide dismutase, which are associated with increased longevity *in vivo*, likely through mitigating the increased levels of ROS generated through increased mitochondrial OXPHOS activity (45, 46). IL-15 has also been shown to promote activity of mTOR in memory CD8+ T cells, which then initiates cell cycle entry and drives migration of T cells to the mucosa where they form a tissue-resident population (47, 48). These IL-15-driven metabolic effects prepare CD8^+^ memory T cells for rapid activation and proliferation during infection (44, 48, 79). Studies have suggested that IL-21, like IL-15, promotes FAO, mitochondrial biogenesis, and production of antioxidants in CD8+ T cells and total CD3+ T cells (49, 50), as well as promoting mTOR activity in TRegs (51).

There is growing evidence for the use of IL-21 and IL-15 to condition T cells prior to adoptive T cell therapy in cancer, since the metabolic adaptations they induce prolong cell survival in mouse models (50, 80). Specifically, these studies highlight a Tscm phenotype of CD8^+^ T cells induced by IL-15 and IL-21, similar to that also described in IL-7-treated T cells (43). However, in this case the T cells show reduced mTOR activity and Glut1 expression, reflecting the differences between cytokine signaling pathways (80) (**Figure 1**). In support of the IL-7-induced metabolic profile, one study showed that high IL-7R and c-Myc expression in CD8^+^ T cells are predictive of survival after adoptive transfer (81). Both IL-15 and IL-21 increase mitochondrial capacity and reduce oxidative stress in T cells, delaying the expression of exhaustion markers such as PD-1 and LAG-3 (49, 50, 80).

Conversely, in some pathologies the metabolic changes driven by these cytokines may be unhelpful. For example, IL-7 is pathogenic in T cell acute lymphoblastic leukemia *via* its role in promoting Pi3K/Akt/mTOR signaling in malignant cells, causing increased proliferation and survival (82, 83). Here, IL-7R blockade is a viable therapeutic approach for patients (83). Additionally, studies have shown that both IL-7 and IL-15 contribute to the persistence of a HIV reservoir in T cells, often resistant to antiretroviral therapy (ART) (84–87). A mechanistic basis for this was recently identified, involving firstly mTOR-mediated expansion of the deoxyribonucleotide triphosphate (dNTP) pool available for viral reverse transcriptase activity, through concerted increases in activity of nutrient uptake and activity of nucleotide biosynthesis pathways, and secondly increased acetyl-coA abundance leading to acetylation/stabilisation of microtubules, facilitating cytoplasmic transport of viral products (88). Consistently, inhibition of both Pi3K signaling and Glut1 abrogate this effect (86, 87, 89). Despite these observations, other studies suggest that IL-7 therapy in HIV patients is nonetheless beneficial in restoring T cells numbers after ART (90, 91), linked to increased mTOR activity in HIV-specific CD8^+^ T cells (92). Understanding the metabolic effects driven by all γc cytokines in T cells may therefore reveal new therapeutic targets for developing new and improved treatments in infection, cancer, and autoimmunity.

### Inflammatory Cytokines

Evidence for the role of inflammatory cytokines in altering T cell metabolism, especially in a disease context is more limited. However, several studies have begun to investigate these effects and provide evidence to warrant further work into understanding the role of cytokine-mediate metabolic effects on T cells in pathogenesis of disease (**Table 1**).

#### IFN-γ

In one study, it was shown that IFN-γ could compensate for the presence of a weak TCR signal by activating mTOR, *via* STAT1 induction, to maintain the production of effector CD8+ T cells (52). Many studies have reported the role of IFN-γ in metabolic reprogramming of other cell types including innate immune cells (93, 94). However, little else is known about the impact of IFN-γ on T cell metabolism. IFN-γ is crucial for Th1 differentiation and induction of IFN-γ release but exerts its own self-limiting feedback loop causing downregulation of IFN-γRII (95). In other subsets, IFN-γ inhibits the differentiation of Th2 and Th17 cells but has been shown to promote TReg and antigen-specific memory T cell generation, often *via* STAT1-dependent mechanisms (96, 97). Future studies may investigate whether IFN-γ-dependent mTOR induction has a role in the pleiotropic effects of IFN-γ on T cells.

#### IL-1β and IL-23

Both IL-1β and IL-23 are essential cytokines for the differentiation of human Th17 CD4+ T cells and are often elevated in inflammatory disease, shifting the balance of the TReg/Th17 axis (98–101). IL-1β signals through mTOR to induce HIF1-α expression and upregulation of glycolysis-related genes, such as Glut1 and HK2 (53–56). This drive towards a glycolytic phenotype is reported to inhibit TReg differentiation and promote Th17 cell induction, due to their differing metabolic requirements (54, 102). For example, in one study *in vitro* TReg differentiation was subverted by the presence of IL-1β, which induced a significant increase in mTOR activity and HIF1α expression, and a decrease in FoxP3 dependent on both of these (54). However, in this case IL-1β did not cause clear effects on TReg oxidative or glycolytic activity, as directly measured by extracellular flux analysis (54). Differences in FoxP3 expression may however have obscured this, since FoxP3 is reported to inhibit glycolysis whilst driving OXPHOS (103). Another study was able to show by extracellular flux analysis that IL-1β increased glycolysis in Th17 cells that had been differentiated for 48 hrs *in vitro* (55) (**Figure 1**). Naïve T cells appear the most susceptible to the metabolic changes mediated by IL-1β and there is evidence to suggest IL-1β can further increase glycolysis in differentiated Th17 cells (54, 55). The functional consequences on effector and memory Th17 cells, and *in vivo* relevance of this effect is yet to be established, but such studies raise the possibility of targeting cytokine signaling and/or metabolic pathways in inflammatory T cell subsets to restore their normal immune function.

IL-23 acts in concert with IL-1β to further increase Th17 cell differentiation and IL-23 is credited as the main driver of a pathogenic Th17 cell phenotype (99). Notably, IL-23 is particularly required in later stages of murine Th17 differentiation, where it promotes mTOR activity. However, the full mechanism of IL-23-induced Th17 cell pathogenicity is not understood and it is unclear if this includes metabolic effects. An integrative phosphoproteomics study identified several unique IL-23R phosphorylation targets in a lymphocytic cell line, including PKM2, a key glycolytic enzyme (57). Phosphorylated PKM2 accumulated in the nucleus where it could then phosphorylate STAT3. Upregulation of several glycolytic genes were seen in the IL-23-treated cell line and it was suggested that IL-23 may modulate metabolism through PKM2 phosphorylation (57). However, a more recent study in primary murine Th17 cells also reported that PKM2 located to the nucleus to increase STAT-3 activity, but found in this case that PKM2 was dispensable for Th17 cell metabolic homeostasis (104). One study using human CD4^+^ T cells showed that IL-1β and IL-23 were sufficient to induce metabolic reprogramming in Th17 cells, which was blunted by CD28 signaling (58) and it has also been shown that differentiating Th17 cells upregulate SIGIRR, an inhibitor of IL-1R, to limit mTOR signaling (53). These studies suggest several mechanisms exist which limit Th17 differentiation through regulation of IL-1β/IL-23-induced glycolysis. As highly pathogenic Th17 cells exhibit increased levels of glycolysis, further studies may investigate potential perturbations of these mechanisms in disease.

#### TNF-α

TNF-α is a highly inflammatory cytokine with aberrant expression in autoimmunity, making it a promising target in disease. Anti-TNF-α biologics are used in rheumatoid arthritis (RA) with varying success, but for other autoimmune diseases such as multiple sclerosis, anti-TNF-α therapy has been shown to exacerbate disease. Mechanisms underlying this are not fully understood and further insight would inform the rational and effective design and use of these therapies (105). Whilst TNF-α has a broad range of cellular targets, recent studies indicate a key role promoting T cell differentiation and function, since blocking TNF-α signaling impairs CD4^+^ T cell maturation and increases the percentage of cells expressing the anti-inflammatory cytokine IL-10 (106, 107). Whether TNF-α impacts T cell metabolic activity remains largely unexplored.

Several studies have shown that T cells chronically stimulated with TNF-α exhibit poor calcium responses to TCR signaling, particularly through impaired calcium/NFAT pathway control of store-operated calcium entry (108). In RA patients, peripheral T cells have defective calcium signaling in response to TCR stimulation (109), which may be attributable to the high circulating serum levels of TNF-α. PBMCs from the RA synovium have also shown defects in calmodulin, a calcium-sensing protein, which can be recovered with anti-TNF-α treatment (110). In contrast, chronic TNF-α stimulation *in vitro* was shown to cause overactivation of Ras/ERK signaling and increased production of ROS (108), an effect similarly seen in T cells from the RA synovium (111). Calcium signaling and ROS levels control multiple aspects of T cell metabolism at each stage of development and within subsets, which are essential for T cell survival and effector function (112, 113). In particular, one study showed that calcium signaling could control pathogenic Th17 cell function through regulation of OXPHOS and oxidative stress (114). TNF-α can act as a co-stimulatory molecule during TCR activation, priming cells *via* a non-redundant mechanism only partially recapitulated by CD28 (115–117). As calcium/NFAT signaling is essential for T cell metabolic reprogramming and proliferation upon TCR activation (118), this may also warrant further investigation into the short-term impacts of TNF-α and anti-TNF-α on T cell metabolism. One study has shown subtle changes in the metabolic profile of CD4^+^ T cells after 24 hr *in vitro* treatment with anti-TNF-α, providing evidence for possible effects but requiring further studies to corroborate this result (59). RNA-sequencing analysis from a study on human memory CD4^+^ T cells after 3 days of activation in the presence of TNF-α or anti-TNF-α, showed multiple differentially expressed genes associated with metabolic processes including HK2, PKM2, and TNFAIP3, a protein that has been shown to limit mTOR signaling and promote autophagy in murine naïve CD4^+^ T cells (60, 119). Further studies into the metabolic actions of TNF-α on CD4^+^ T cells and how these may be targeted in disease, could reveal novel therapeutic opportunities.

### Anti-Inflammatory Cytokines

There is less evidence for the roles of anti-inflammatory cytokines on T cell metabolism. IL-10 for example is described to modulate metabolic reprogramming in B cells and macrophages (120, 121), but little is known about its effects on T cell metabolism. TGF-β has however been explored in more depth, which is discussed below (**Table 1**).

#### TGF-β

TGF-β signaling has been shown to inhibit mTOR activity in CD4^+^ T cells, through Smad3-mediated inhibition of Pi3K/Akt activity (61), limiting downstream induction of key glycolytic molecules such as Glut1 and HK2 (61, 62). TGF-β signaling also induces T cell polarization to an inducible (i)Treg phenotype, at least in part mediated by its suppression of mTOR and favoring of an OXPHOS-driven Treg metabolic profile (29, 62, 63, 102). Compared to iTregs, thymic-derived (t)Tregs exhibit higher glycolysis (32, 62). One study was able to show that culturing iTregs with TGF-β inhibited glycolysis *via* the Pi3K/Akt/mTOR pathway and reduced their suppressive capacity (62). In contrast, studies previously have shown that increasing Pi3K/Akt/mTOR signaling in Tregs promotes proliferation at the expense of suppressive capacity (30). Although the mechanism of this distinction in unclear, these data highlight the differing functional responses of T cells to the same cytokine-driven metabolic effects. TGF-β is a promising target in cancer immunotherapy due to its suppressive activity on tumor cells, however, its suppression of effector T cells can also dampen anti-tumor activity. A recent study showed that TGF-β present in the tumor microenvironment could inhibit OXPHOS in CD4^+^ effector T cells *in vitro* (63) (**Figure 1**). Mechanistically, this was mediated *via* phosphorylation of Smad proteins, which then localized to the mitochondria, associated with decreased activity of ATP-synthase. Selectively alleviating the immuno-suppressive effects of TGF-β on immune cells whilst maintaining the anti-tumor effects would be an important development in cancer therapy. Further studies are required to investigate the metabolic changes induced by TGF-β and how to target these to recover T cell effector function. TGF-β levels in autoimmunity are often insufficient (122, 123). However, due to its multitude of immuno-suppressive effects, treating with TGF-β can be quite toxic (122). The effect of TGF-β on T cell metabolism in an inflammatory disease context is largely unexplored. Understanding the mechanisms of cytokine-mediated metabolic reprogramming in T cells may lead to new therapeutic targets in selectively modulating metabolic pathways in order to control T cell pathogenesis, an approach that has shown promise in studies so far (124).

## Hormones

### Adipokines

#### Leptin

**Figure 2:**
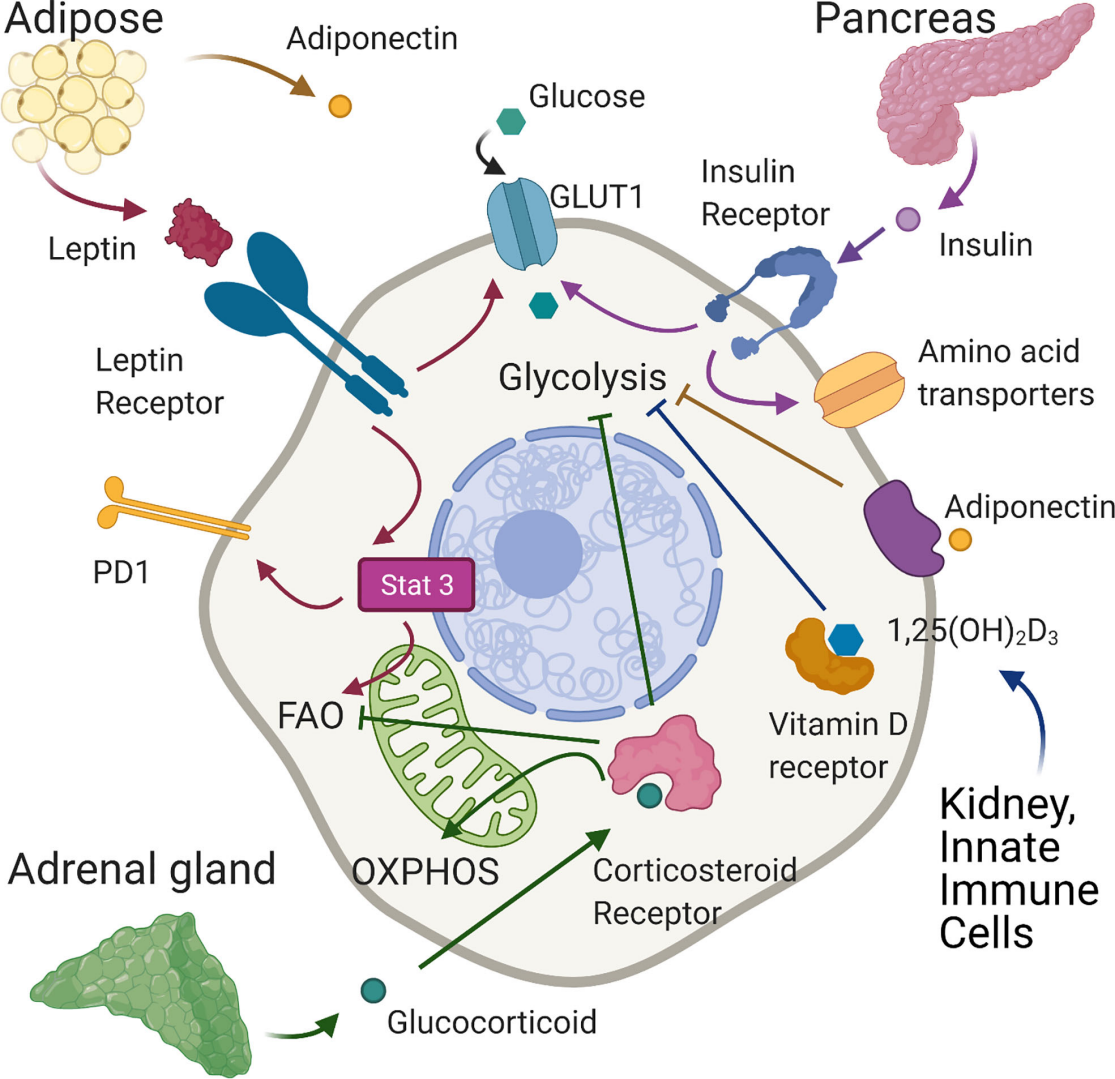
Effects of hormones on T cell metabolism. In normal conditions leptin, produced mainly by adipose tissue, binds the leptin receptor on T cells, upregulating GLUT 1 expression on CD4+T cells enabling the shift towards glycolysis required for effector function. In tumour models of diet-induced obese mice, leptin signaling induced-phosphorylation of STAT3 upregulates PD1 expression and FAO in tumour-infiltrating CD8+ T cells leading to decreased CD8 effector T cell function and tumour progression. Adiponectin, also produced largely by adipose tissue, is able to inhibit glycolysis and T cell inflammatory activity in CD4+ cells. Insulin, secreted by pancreatic islets, binds insulin receptors expressed on activated T cells increasing glycolysis and the expression of amino-acid transporters. Corticosteroids, released by the adrenal gland, can suppress glycolysis and increase OXPHOS. In tumour immunity, corticosteroid sensitive, low affinity CD8 T cells are inhibited by corticosteroids through impairment of fatty acid oxidation. Vitamin D, generated in the skin and then activated mainly in the liver and kidneys may reduce inflammation by suppressing glycolysis in T cells. Created with Biorender.com.

Similar in structure to cytokines, adipokines are peptide hormones produced largely by adipose tissue that can integrate systemic metabolism with immune function. The best-described, leptin is secreted predominantly by adipocytes (125) and is engaged in body weight homeostasis and satiety (126). Leptin levels are generally stable and indicate overall energy availability (126), being proportional to fat mass and body mass index (BMI) (127, 128). Consequently, hypoleptinemia is seen in states of malnutrition (129) whilst obesity is characterized by hyperleptinemia and leptin desensitization (130, 131). However, leptin levels also increase acutely during infection, irrespective of BMI (132). An indication of its role in the immune system, the leptin receptor (LepR), a member of the class I cytokine receptor family, is expressed on all immune cells (133, 134) and is upregulated on T cells upon activation (135, 136). Consistent with this, defective T cell activation and function occur upon leptin deficiency and in malnutrition (64, 137–140), which can be rescued by leptin treatment *in vitro* or *in vivo* (64, 137–139). Leptin binding to LepR induces receptor dimerization, autophosphorylation of JAK2 and phosphorylation of four tyrosine residues in the LepR cytoplasmic tail (141). This leads to activation of the Pi3K/Akt and the MAPK signaling pathways and to phosphorylation of STAT3. Subsequent STAT3 dimersation and translocation to the nucleus activates transcription of target genes (142–145). A direct effect of leptin on immune metabolism was first demonstrated by Saucillo et al. (64). Here, food deprivation in mice induced persistent defects in effector CD3+ T cell function, which were accompanied by metabolic impairments –specifically diminished glucose uptake and glycolysis upon stimulation, linked to the failure to upregulate Glut 1 expression. These effects could be rescued by either *in vivo* or *in vitro* leptin treatment demonstrating that leptin could indicate nutritional status to activated T cells ‘licencing’ them for the metabolic changes associated with effector function (**Figure 2**). Subsequent studies in a mouse model of experimental autoimmune encephalomyelitis (EAE) (65) indicated that leptin deficiency inhibited glycolysis in Th17 cells but not TRegs, leading to reduced disease severity. This was associated with decreased levels of HIF-1α mRNA and protein in Th17 but not Tregs and established the potential for manipulating this axis to control T cell metabolism and dysregulated activity in autoinflammatory disease. Conversely, others have examined whether increased leptin activity could potentially augment or restore T cell function in the context of anti-tumor immunity. In one study, melanoma-infiltrating CD8+ T cells were found to exhibit higher LepR levels than cells present in lymphoid tissue, and tumors engineered to express leptin grew more slowly, leading to improved survival. This effect required T cell LepR expression and was associated with significant increases in CD8+ T cell mitochondrial capacity and anti-tumor cytokine expression. Importantly from a therapeutic perspective, the effects could be recapitulated with a modified oncolytic vaccinia virus encoding leptin and injected directly into poorly immunogenic melanoma and pancreatic tumor models, which conferred a significant survival advantage (66). Notably in this study, oncolytic virus treatment showed no benefit in obese mice, where leptin levels are already likely to be high. Consistent with this, Wang et al. (146) linked high leptin levels in mice with diet induced obesity (DIO) and in obese humans to increased frequencies of dysfunctional T cells with high immune checkpoint (i.e. PD-1) expression. In this study, grafted B16 melanoma grew faster in obese mice, but those with LepR deficient T cells were better able to control tumor growth, associated with reduced PD-1 expression on CD8^+^ T cells. The link between leptin signaling and PD-1 expression was found to be STAT3. As noted earlier, this mediates leptin signaling, but also drives PD-1-expression (147). Links between T cell function, STAT3 and PD-1 were also shown in an obese mouse breast tumor model by Zhang et al. (67). Leptin and PD-1 ligation in this setting increased STAT3 activity in tumor-infiltrating T cells, leading to decreased glycolysis and increased FAO. CPT1a, the critical and rate limiting enzyme of FAO is also direct target of STAT3 (148) and STAT3 signaling and switching of CD8+ T cell effector metabolism from glycolysis to FAO was associated with decreased effector T cell numbers and function and more aggressive breast tumor development (**Figure 2**, **Table 1**).

#### Adiponectin

Adiponectin is another peptide hormone or adipokine secreted largely by adipose tissue. However, contrary to leptin, adiponectin levels generally inversely correlate with BMI (149). Adiponectin mediates direct metabolic effects on skeletal muscle, vascular endothelium, adipose tissue, the pancreas and liver. Circulating levels negatively correlate with markers of inflammation in metabolic syndrome but positively correlate with insulin sensitivity. These findings indicate adiponectin may regulate the metabolic and inflammatory activity of immune cells, but this has not been studied in detail so far. One report has however identified that adiponectin can regulate inflammatory cytokine production and metabolic activity of T cells. In this study, CD4^+^ T cells from mice fed a high fat diet (HFD) spontaneously expressed higher levels of IFN-γ and TNF-α than those on a control diet, which was suppressed by treatment with adiponectin *in vitro*. Additionally, neutralization of adiponectin *in vivo* during a course of treatment with filarial adult worm extract increased circulating frequencies of IFN-γ^+^ cells. Taken together these data indicate that adiponectin regulates CD4^+^ T cell inflammatory activity, which the authors further show may be linked to direct inhibitory effects on glycolysis (68). Adiponectin binds to specific GPCRs, AdipoR1 and AdipoR2 to elicit downstream signaling events. Specifically, ligation recruits the adaptor proteins APPL1 and APPL2, which interact with other signaling pathways resulting in the phosphorylation of Akt, MAPK and AMPK and increased activity of PPAR-α. Additionally, AdipoR1 ligation augments insulin signaling, since APPL1 directly interacts with insulin receptor substrates (IRS) (149). In T cells it appears that some effects of adiponectin are mediated by AMPK, whilst others are AMPK-independent (68) (**Figure 2**, **Table 1**).

### Insulin

Insulin is a peptide hormone well described to control the systemic metabolism of carbohydrates, proteins and lipids through direct promotion of glucose uptake and storage, as glycogen or lipids, by skeletal muscle, liver and adipose tissues. However, its receptor is expressed on many cell types, including immune cell subsets. Receptor binding induces its dimerization and autophosphorylation, leading to phosphorylation of IRS. The signaling cascades downstream of these signals overlap with those induced by TCR/CD28 signaling and include the MAPK and Pi3K/Akt/mTOR pathways. When we consider the metabolic changes associated with T cell activation it is perhaps not surprising that insulin has now been shown to have a role. The insulin receptor is not detectable on resting T cells but is upregulated within 24 hours of T cell activation (69). Both CD4^+^ and CD8^+^ T cells lacking insulin receptors were found to be dysfunctional, demonstrating reduced proliferation and inflammatory cytokine production compared to control cells when stimulated *via* the TCR *in vitro. In vivo*, after influenza infection, mice with insulin receptor deficient T cells had much more severe disease, fewer antigen-specific CD4^+^ and CD8^+^ T cells and fewer cells producing IFN-γ and TNF-α. The functional deficiencies T cells lacking insulin receptors were underpinned by metabolic dysfunction. Cells had lower expression of c-Myc, Glut transporters and key glycolytic enzymes including HK2 and LDHA. The expression of amino-acid transporters was also decreased and the cells demonstrated reduced rates of oxygen consumption and lactate production. Consistently, insulin treatment of receptor-replete activated T cells increased their activation, proliferation, cytokine production and metabolic activity (69) (**Figure 2**, **Table 1**).

## Steroid Hormones

Alongside the peptide hormones/adipokines described above, the other class of hormones with reported effects on T cell metabolism and function include the steroid hormones - specifically glucocorticoids and the active form of vitamin D.

### Glucocorticoids

Glucocorticoids, also known as corticosteroids are steroid hormones released by the adrenal gland according to circadian rhythm to regulate feeding and sleep-wake cycles. Glucocorticoids are also released following stress caused by infection or inflammation where they contribute to the restoration of homeostasis through their potent anti-inflammatory functions (150, 151). Glucocorticoids bind the glucocorticoid receptor, which is expressed by almost all cell types including immune cells and can have wide reaching effects on gene expression (152) regulating cell metabolism, growth, differentiation and apoptosis. Their systemic metabolic effects are related to lipid and glucose homeostasis, specifically they stimulate lipolysis in adipocytes and induce mobilization of extra hepatic amino acids and stimulate gluconeogenesis in the liver while inhibiting glucose uptake in muscle and adipose tissue (150, 151). Therapeutically, glucocorticoids are widely used to treat many inflammatory conditions such as rheumatoid arthritis, asthma, sepsis and multiple sclerosis (150, 151) as well as hematopoietic malignancies such as leukemia. In this context they are reported to inhibit cell cycle progression and increase apoptosis (153) but also to exert metabolic effects including disruption of glycolysis (154–156) and increasing mitochondrial oxidative phosphorylation (157). Corticosteroids are also used to suppress immune-related adverse events experienced by cancer patients treated with immune checkpoint blockade. In this context, Tokunaga et al. (70) showed that sensitivity of CD8 +T cells to corticosteroids is proportional to the levels of TCR stimulation and is associated with changes in their metabolic capacity. Specifically, low affinity T cells were more sensitive to the inhibitory effects of corticosteroids, which may be explained by the fact that the glucocorticoid receptor can be phosphorylated by ERK1/2 and JNK downstream of the MAPK pathway preventing translocation into the nucleus. Indeed, higher affinity cells demonstrated greater phosphorylation of the glucocorticoid receptor, and less sensitivity to corticosteroids. There was a significant enrichment of genes involved in fatty acid metabolism upon corticosteroid treatment of low affinity CD8+ T cells and FAO was impaired in these cells (**Figure 2**). Therefore, corticosteroid treatment during immune checkpoint blockade may impact the response to low affinity tumor antigens by suppressing fatty acid metabolism of these cells. Notably, it was recently shown that glucocorticoids are synthesized locally in tumor microenvironments by various cell types including myeloid lineages, macrophages and T cells themselves, meaning that their effects on T cell metabolism and function may be particularly relevant in the context of solid tumors (158, 159) (**Table 1**).

### Vitamin D

The active form of vitamin D_3_, 1,25-dihydroxyvitamin D_3_ (1,25D), is a strong immunomodulator, able to suppress inflammatory T cells and promote the generation of TReg (160). Vitamin D deficiency and mutations in the vitamin D receptor (VDR) gene are associated with increased risk of multiple autoimmune diseases, making vitamin D_3_ therapy a promising candidate. However, clinical applications have shown limited success so far (160). 1,25D, which is generated largely in the kidneys but importantly also by activated innate immune cells (160) and in malignant tissues (161), primarily acts by binding to the VDR, which forms a heterodimer with the retinoid X receptor (RXR) to regulate transcription of genes (162). Some studies have begun to highlight a role for 1,25D in regulation of T cell metabolism, but more functional studies are lacking. Work by Calton et al. (163, 164) outlined a correlation between low serum vitamin D_3_ level and high metabolism in PBMCs, as well as an increase in production of inflammatory cytokines. In several studies, 1,25D has been shown to decrease gene expression of key metabolic regulators such as c-Myc and members of the Pi3K/Akt/mTOR pathway (71–73). One study, although using a low number biological replicates, showed that 1,25D treatment reduced mTOR activity in CD4^+^ T cells from an EAE model (74). Overall, these data begin to suggest that 1,25D may suppress glycolysis in T cells (**Figure 2**). An effect that could act as an additional mechanism in driving TReg polarization (102). In addition, another study in EAE showed that 1,25D induced upregulation of the enzyme required for production of methionine, BHMT1, which then increased global DNA methylation levels in CD4^+^ T cells and promoted conversion to Tregs (75). There are some effects of 1,25D that cannot be fully explained by changes in transcriptional regulation. For example, occasionally a reduction in inflammatory cytokine production but not gene expression is seen in 1,25D-treated T cells (165). Further studies are required to understand the potential non-genomic roles of 1,25D, as reviewed in other cell types previously (166, 167), in controlling cellular metabolism and the resulting impact on T cell function in disease. Better understanding of the mechanisms of immunosuppression by 1,25D will allow for improved therapeutic approaches. For example, in combination with metabolic modulators (**Table 1**).

## Summary

In summary, dynamic and coordinated changes in T cell metabolic pathway activity directly determine their immune function and consistently their role in promoting or resolving disease. These changes are initiated by signals arising from their TCR and co-stimulatory receptors, but importantly are further regulated by multiple additional inputs from the local tissue microenvironment, or that fluctuate systemically in response to the host’s nutritional and/or disease status. In addition to the well-described effects of changes in nutrient or metabolite availability, we have summarized here the emerging evidence that cytokines and hormones act as additional modulators of the T cell metabolic phenotype, which may be particularly relevant in diseased tissue environments or pathologies where their systemic levels become dysregulated. Consequently, targeting such pathways, either alone or alongside direct metabolic manipulation may lead to novel therapeutic approaches to restore dysfunctional T cell metabolism in chronic disease.

## Author Contributions

ELB, NG and SD wrote and edited the manuscript. All authors contributed to the article and approved the submitted version.

## Funding

SD is funded through a European Commission Marie Sklowdowska Curie Individual Fellowship and a Leukaemia UK John Goldman Fellowship. NG is funded through a Wellcome Trust ISSF award and EB is funded through the Wellcome Trust MIDAS doctoral training programme at UoB.

## Conflict of Interest

The authors declare that the research was conducted in the absence of any commercial or financial relationships that could be construed as a potential conflict of interest.
